# Home-Cage Training for Non-Human Primates: An Opportunity to Reduce Stress and Study Natural Behavior in Neurophysiology Experiments

**DOI:** 10.3390/ani15091340

**Published:** 2025-05-06

**Authors:** Francesco Ceccarelli, Fabrizio Londei, Giulia Arena, Aldo Genovesio, Lorenzo Ferrucci

**Affiliations:** 1 Department of Physiology and Pharmacology, Sapienza University of Rome, Piazzale Aldo Moro 5, 00185 Rome, Italy; francesco.ceccarelli@uniroma1.it (F.C.); fabrizio.londei@uniroma1.it (F.L.); giulia.arena@uniroma1.it (G.A.); 2 Institute of Biochemistry and Cell Biology (IBBC), National Research Council of Italy (CNR), Via Ramarini 32, Monterotondo Scalo, 00015 Rome, Italy; 3 Behavioral Neuroscience PhD Program, Sapienza University, 00185 Rome, Italy; 4 Department of Pharmaceutical Sciences, University of Piemonte Orientale, 28100 Novara, Italy

**Keywords:** non-human primates, home-cage training, automated cognitive testing, natural behavior, free-moving recording, wireless recording

## Abstract

Research involving non-human primates remains a cornerstone in fields such as biomedical research and systems neuroscience. However, the daily routines of laboratory work can induce stress in these animals, potentially compromising their well-being and the reliability of experimental outcomes. To address this, many laboratories have adopted home-cage training protocols to mitigate stress caused by routine procedures such as transport and restraint—a factor that can impact both macaque physiology and experimental validity. This review explores the primary methods and experimental setups employed in home-cage training, highlighting their potential not only to address ethical concerns surrounding animal welfare but also to reduce training time and risks for the researchers. Furthermore, by combining home-cage training with wireless recordings, it becomes possible to expand research opportunities in behavioral neurophysiology with non-human primates. This approach enables the study of various cognitive processes in more naturalistic settings, thereby increasing the ecological validity of scientific findings through innovative experimental designs that thoroughly investigate the complexity of the animals’ natural behavior.

## Simple Summary

Studies using non-human primates help us better understand cognitive functions, yet traditional experimental laboratory methodologies may have as a side effect a significant time demand for research staff and the induction of stress in the animals, potentially compromising animals’ welfare and experimental validity. This review discusses methods called “home-cage training”, an approach where non-human primates participate in experimental testing within their usual living environment. In this review, we will show how the home-cage approach minimized the research staff’s involvement in the experiments’ handling and the stress induced by transportation and restraint, improving animal welfare and the reliability of experimental results compared to the classical experimental approach. This innovation significantly enhances the quality and ethical standards of neuroscience research, offering valuable insights into cognitive functions related to human behavior.

## 1. Balancing Animal Welfare and Scientific Validity: Managing Stress in Laboratory-Housed NHPs

Stress significantly influences the efficiency with which individuals handle daily challenges. While moderate stress can enhance performance by increasing focus and motivation, excessive or prolonged stress often impairs our performance in cognitive and behavioral tasks, whose neural correlates are investigated in biomedical research and systems neuroscience. Since these research fields still rely to a great extent on the use of animal models, especially non-human primates (NHPs), it thus becomes crucial for researchers to pay particular attention to the physical and psychological welfare conditions of animals living within research facilities, mainly for two reasons. The first of these is of an ethical nature. The 3Rs principle (replace, reduction, and refinement), first developed by Russell and Burch over 60 years ago [1], sets out a series of ethical guidelines for the use of animals in research, and is now used as a starting point in several countries, such as the UK, EU, and USA, to regulate and legislate the use of animals for scientific purposes. In particular, the term refinement refers to the effort to maximize animal welfare by minimizing pain, suffering, and distress. This last point is of crucial importance for animals living in laboratory settings because it involves special attention not only to their physical conditions but also to their psychological ones.

**Figure 1:**
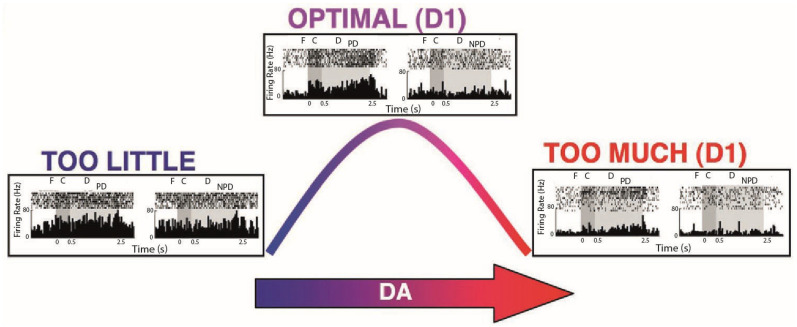
Effects of dopamine release in prefrontal circuits on the discharge activity of a dorsolateral prefrontal cortex neuron during a delayed spatial memory task. Limited (left box) or excessive (right box) stimulation of D1/5 receptors leads to loss of tuning for the preferred direction (left rasters in each box) compared to the non-preferred direction (right rasters in each box). An adequate level of stimulation (top central box) contributes to spatial coding by suppressing the firing rate for the non-preferred condition. Adapted from [18].

The second reason is linked to the first: taking care of the ethical aspect and psychological well-being of animals can also guarantee the success of experimental procedures, ensuring the validity and replicability of scientific experiments using animals. Stress has been defined as ‘the nonspecific response of the body to any demand made upon it’ [2]. In itself, stress does not necessarily have a negative connotation, as the common language seems to suggest, but rather concerns a constant physiological condition to which our organism is subjected, but which must be kept within certain ranges to allow us to function at our best [3,4,5,6]. Both low and high levels of stress can be detrimental to performance, whereas moderate activation leads to optimal performance, with an inverted U-shaped relationship. In lab settings, the main risk is that non-human primates may be subjected to high levels of stress, exhibiting both acute and chronic stress responses, which may alter various fundamental cognitive processes that depend on the proper functioning of the prefrontal cortex [7]. In fact, this ‘inverted U’-shaped relationship between stress and performance is also found in the relationship between the release of some neurotransmitters, such as catecholamines, in the prefrontal cortex (PFC), especially noradrenaline (NE) and dopamine (DA), and performance in different cognitive tasks [8,9,10]. NE and DA, produced by neurons of the brainstem and basal ganglia, are released to a greater extent in the PFC following stressful events [11,12,13]. The excess of these neurotransmitters in prefrontal circuits, for example, has been observed to have the effect of suppressing firing and altering the spatial tuning of dorsolateral PFC neurons (Figure 1) during a delay period in a spatial working memory task [14,15,16,17,18,19]. More generally, many studies with animal models have shown how stress and cholinergic stimulation of the PFC extensively affect various prefrontal functions, influencing processes of decision-making [20], cognitive and behavioral flexibility [21,22], encoding of task rules [23,24], and working memory in general [25], through molecular mechanisms that alter brain function at the intracellular level, affecting the organization of cortical networks and functional connectivity [26,27,28].

For these reasons, stress management during the laboratory daily routine becomes crucial for scientists working with NHPs, especially for those interested in studying the neurophysiology of higher cognitive functions. Husbandry procedures thus represent a major source of stress for monkeys [29,30]. In these procedures, animals are daily subjected to events that, although physically non-harmful, can be potentially stressful, such as social isolation, veterinary procedures, or moving from one cage to another. In order to keep the stress response under control, daily training plays an essential role. Positive reinforcement training is the standard method for teaching NHPs to cooperate with husbandry procedures [31,32,33,34,35]. It has proven effective even for complex and potentially stressful procedures such as pole-and-collar training [36] or the use of restraint chairs [37,38], which are commonly employed in behavioral neurophysiology experiments. Further efforts have been made to minimize invasive procedures, i.e., those requiring direct intervention on the animal’s body. For example, the use of head-holding devices that are secured to the skull with surgical procedures is necessary for different experimental protocols. Non-invasive head-holding systems have been developed for this purpose using systems that allow head restraint while minimizing stress for the animals and that do not require surgical implants [39,40,41,42,43]. These systems allow the collection of eye movements, fMRI data acquisition, or the recording of neural activity with data acquisition systems that require cables. In recent years, a growing body of literature has been investigating new methods to optimize and improve the training experience of NHPs used in laboratories. To this end, an increasing number of laboratories have started to rely on training systems directly in the home-cage. As we shall see in the next Section, home-cage training has several advantages over more traditional forms, such as more effective stress management, which is important for the reasons mentioned above, but also opens up the possibility of investigating new experimental questions that would be more difficult to investigate in a classical setup. In the final Section, we will illustrate how refinements in cellular recording techniques within the home-cage have given rise to innovative ethological approaches for probing cognitive mechanisms.

## 2. The Evolution of Home-Cage-Based Methods for NHP Training

As introduced previously, NHP experiments in traditional and modern behavioral neurophysiology generally involve daily procedures conducted by experimentalists and technicians to train animals and subsequently carry out behavioral tasks critical to ongoing research. NHPs are typically placed into a specialized primate chair using capture techniques established and endorsed among the laboratory communities. These procedures often require the combined usage of neck collars, poles, and cage-based squeezing mechanisms designed to facilitate the animal’s cooperation and guide its movement into the primate chair. Various techniques have been proposed to refine these procedures, employing positive or negative reinforcement strategies and habituation training to encourage desired behaviors and reduce stress-related responses [36,37]. Alternative approaches have also been developed to encourage NHPs to spontaneously approach and occupy the chair [44]. Nevertheless, these methods require protracted and day-to-day training periods, which significantly increase staff workloads. Furthermore, despite observable reductions in behavioral stress indicators, physiological stress responses might persist [45], which is an important confounding factor for interpreting data derived from experimental studies. Following chairing procedures, animals are usually separated from their cage mates and transported to a dedicated experimental room containing task-related apparatuses. Depending on the specific chair design employed [37,46,47], varying degrees of restriction for the NHP’s range of movements are imposed to facilitate the accurate monitoring of behavioral variables, such as eye and hand movements, interactions with manipulable devices (e.g., joysticks, buttons), and ultimately for neuronal signal recording. Collectively, social isolation and physical restriction may significantly impact NHPs’ well-being and chronic stress levels, potentially compromising their health and behavior, while at the same time negatively influencing the experimental outcomes.

### 2.1. Historical and Technological Milestones in Non-Invasive Cognitive Testing Approaches for NHPs

**Figure 2:**
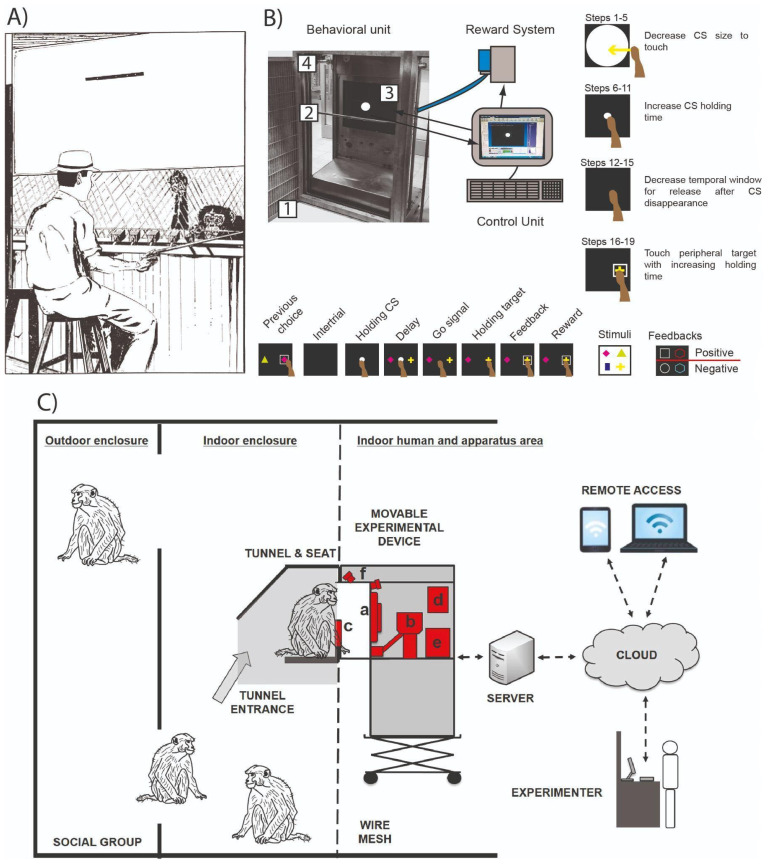
(**A**) Illustration of the seminal setup of Spence [48], apparatus for the discrimination task of associative problems, located alongside the fence of the chimpanzee enclosure. Representation of the experimenter, controlling through a manipulator the presentation of problem pairs on a trial-by-trial basis. Adapted from [48]. (**B**) Home-cage setup proposed by Sacchetti and colleagues [95]. On the top left, the chassis of the behavioral unit is attached to the NHP cage (1). Embedded in the chassis are an adjustable reward pipe (2), a touchscreen (3), and a camera (4) for monkey monitoring. The unit is wired with a liquid reward system, which, together with the touchscreen, is operated by the computerized control unit used for task presentation and behavioral data collection. On the right, an illustration of the successive non-match-to-goal learning steps is displayed on the screen. At the bottom is the sequence of events occurring in each trial performed by the monkey (events of computer trials not shown, see [95]) of the task and the link with the previous trial, which shows that the animal must continuously consider the previously chosen object and switch to the alternative object option. Adapted from [95]. (**C**) Workflow of the automatized home-cage setup of Fizet and colleagues [88], route to reach the movable apparatus installed next to the cage (a: touchscreen; b: reward system; c: system for primate identification via RFID; d, e: computer system; f: video cameras). Graphical representation of the connectivity with the control unit for computerized automated control of session progression, primate identification, task presentation, data collection and storage, and monitoring via cloud. Adapted from [88].

Despite the widespread reliance on these traditional methodologies, less invasive and restrictive approaches were proposed. These alternative approaches, designed to minimize stress and improve NHP welfare without compromising experimental rigor, constituted the main topic of this part of the review. Research in comparative psychology has historically pursued a divergent experimental approach in contrast to the conventional approach in behavioral neurophysiology. A pioneering study by Spence [48] designed a testing apparatus for chimpanzees housed within colony enclosures for discriminative tasks (Figure 2A). The apparatus was positioned next to the enclosure’s fence and involved the presentation of changeable metal object pairings, which, using underneath boxes, hid food rewards. Chimpanzees could interact with the object pairs without physical restriction, with the experimenter manually placing appropriate new pairs and rewards following a trial-based design. Comparative psychology studies involving chimpanzees and similar testing apparatuses have revealed several cognitive and perceptual processing similarities and differences between humans and non-human primates [49,50,51,52]. Nevertheless, for ethical and practical reasons, behavioral neurophysiology has shifted mainly toward the extensive use of other NHPs, primarily various macaque and marmoset species, which will be the focus of further discussion in this review. The core components of this approach, a direct manipulation of object pairs and no physical restriction for NHPs during task execution, while preserving a structured trial-based setting, have led several NHP research groups to evolve the concept into several variants of the Wisconsin General Test Apparatus (WGTA). In the original WGTA design [53,54], NHPs were placed within a moderately sized test cage, with no physical restraints and free to interact with object pairs presented on a tray containing concealed food rewards, aiming to track down the object associated with the reward. At the opposite side of the apparatus, a workstation occupied by the experimenter controlled the movement of a plate, which was lowered in front of the test cage at the end of each trial to allow the experimenter to control trial progression. Subsequent advancements promoted a progressive automatization, by replacing the manual trial progression system with motorized trays and introducing sensor-based monitoring of NHP movements [55,56,57], culminating in fully automated setups employing visual object projection screens equipped with reward dispensers [58,59,60,61]. These initial setup configurations described so far narrowed scientific investigation to basic associative learning and/or object–pair discrimination paradigms (for a detailed review, see [62]), which reduced the spectrum of behavioral tasks that could be used to test NHPs. Technological advancements subsequently led to the development of a modified setup in which the testing cage remained separated from the animals’ home environment. This arrangement comprised a computer and monitor positioned directly in front of the test cage, facilitating and broadening a flexible design and presentation of cognitive tasks according to the scientific needs. Task interaction was facilitated through the installation of a joystick, allowing NHPs to actively perform and control task execution. The advantage of this setup to manipulate the task features permitted the investigation of behavioral correlates of hemispheric lateralization [63,64], visual–sensory information processing, stimulus categorization [65,66], and associative learning [67].

### 2.2. Home-Cage Systems: Key Aspects of the Current Proposed Approaches and Progressive Advancement Toward Automated Cognitive Testing

While these initial alternative methodologies represented significant advancements over the classical neurophysiological chair approach for NHP welfare, they also had some limitations. The primary limitation was the necessity to move animals from their housing environments to isolated test cages designated specifically for experimental tasks. This involved an additional pre-experimental training phase in which NHPs must be conditioned to respond to the staff’s commands to voluntarily move toward the testing cage or, alternatively, enter into a transport cage needed for displacement. In addition, even brief periods of test cage confinement may represent a stress source, thus potentially constraining the maximum duration and motivation of daily sessions. One approach within a naturalistic context has been to move the observation learning from the chair [68] to the cage [69,70,71], but with no automatic stimulus presentation. A significant advancement was represented by the Language Research Center’s Computerized Test System (LRC-CTS) [72,73,74,75]. The LRC-CTS consisted of a mobile workstation equipped with a computer and a monitor for visual task presentation, a joystick and speaker for task execution and acoustic feedback release, and a food dispenser for trial rewards. This workstation could be attached directly to the NHP’s home-cages, allowing cognitive tasks to be tested continuously throughout the day based on the animal’s voluntary interaction with the apparatus. Studies utilizing this home-cage setup demonstrated successful learning of classical associative problems similar to WGTA [76,77], discrimination of perceptual features [78], quantities paired with numerical symbols [79], and response strategy implementation in maze solving [80].

Recent decades have witnessed significant refinement and consolidation of the home-cage methodologies for neurophysiological experiments, proposing a wide range of solutions to accommodate disparate experimental and structural needs related to the broad range of NHP housing environments. Table 1 encapsulates the key features of the leading behavioral setups devised that represent the current state of home-cage cognitive training and testing for NHPs. Home-cage approaches emphasize the adaptation of experimental setups directly in NHP home enclosures, aiming to optimize animal welfare, reduce human intervention during experimental phases, and provide a more naturalistic approach to behavioral and cognitive testing. The core structure of home-cage setups includes two main functional units: the behavioral unit and the control unit. The behavioral unit integrates components necessary for task presentation and interaction, behavioral signal recording, and reward delivery mechanisms embedded in a backbone frame designed to integrate with the home-cage. The control unit consists of the computerized systems responsible for designing, controlling, and managing task testing and training, collecting multisource data through I/O features, and, in some cases, transmitting data remotely to external systems for external storage and monitoring. Home-cage proposed setups share this basic arrangement but differ significantly regarding their implementation and typology of components. The first differentiation affects the chassis design for the behavioral unit integration, where several home-cage setups use a chassis that replaces removable cage walls found in apposite cage models [81,82,83,84,85,86]. Conversely, other approaches devise a chassis equipped with attachment structures to dock to cages that have holes or bars in the walls [87,88,89,90,91]. Additionally, specific configurations offer cage expansions, forming accessible yet confined spaces connected to home enclosures [92,93,94,95]. Hardware components for task presentation and interaction predominantly include high-impact-resistant touchscreens [81,86,88,89,91,92,95]. In some home-cage setups, touchscreens are replaced by tablets [87,90], hybrid systems combining monitors with joysticks [93], or interactive buttons [94]. Reward delivery is precisely controlled and temporally schedulable, with variations including liquid rewards administered through peristaltic or custom pumps [81,86,87,89,94,95], solid rewards dispensed via food trays [88,90,91,92,93], or both [84]. Control units are generally independent from behavioral units, connected via cables for session control and data acquisition, and positioned separately to facilitate staff management of the apparatus, tasks, and session execution. Exceptionally, Womelsdorf et al. [84] and Martin et al. [91] integrate control and behavioral units into a compact system to encourage a more compact design. Task and session programming involves multiple programming languages and pre-developed routines according to the proposed home-cage setup, often utilizing specialized toolboxes commonly adopted in neuroscience research with NHPs and humans.

The solutions proposed within the home-cage approach not only create an experimental environment beneficial for NHP welfare but also substantially reduce the research staff’s workload during experiment execution. Within this context, one of the most crucial and time-consuming aspects involves the pre-experimental training phases, in which naive animals, or animals with previous experience performing different tasks, must be trained to effectively interact with the experimental apparatus and clearly understand the task rules and phases, thus ensuring appropriate performance. Several proposed HC setups emphasize the ability to automate experimental and training session management [81,84,86,88,89,91,93,95,96,97], thereby minimizing staff involvement typically required during these phases. In a recent study with marmosets [98], the system was implemented to allow different types of progression criteria based on the experimental design. For example, it offered the option to change steps after a predefined number of trials or once a certain number of correct trials were completed within a specific window of multiple trials. Concurrently, these automated systems provide flexible training frameworks capable of adapting to the individual differences observed among NHPs. The rationale underpinning these training protocols involves simplifying the final experimental task by subdividing it into discrete steps. Each step requires the NHP to perform a specific behavioral interaction with the setup while learning and following distinct rules (Figure 2B). Additionally, undesired or alternative behavioral strategies, beyond the targeted responses, are systematically inhibited or extinguished during training [96]. This structured approach subdivides the training protocol into sequential learning steps, typically organized according to increasing levels of complexity. Throughout each training step, the animal’s performance is continuously monitored by the computerized control unit, which, based on threshold criteria predetermined by the experimenter, can automatically advance the animal to subsequent steps or regress to previous ones as necessary. Initial steps in these protocols typically focus on enabling the animal to engage successfully with the behavioral unit and to receive rewards through basic interactions, such as touching variably-sized stimuli presented on a touchscreen, using joysticks, or correctly pressing specific levers and buttons. Subsequent steps diverge among setups according to the complexity and demands of the final experimental tasks, with dedicated training protocols designed for paradigms such as Non- and Match-to-Sample [81,86,88,89,93,95], Change Detection [89], two-choice discrimination [93], Five-Choice Serial Reaction Time Task [88], Self-Ordered Spatial Search [88], Memory-guided pro/anti reach tasks [96], and complex visual search tasks [84]. Insights gained from convergent experiences with these training protocols highlight the importance of dynamically introducing additional sub-steps during training phases where animals exhibit individual learning challenges. Furthermore, adjusting incorrect behaviors to enhance task-related cognitive performance can be encouraged by planning periods of enforced reiteration of the initial or newly introduced steps, based on real-time behavioral monitoring. Intervention strategies, such as those described, can be implemented through the software integrated into various home-cage training systems.

**Table 1:** Studies that developed the leading home-cage setup methodologies for cognitive training and testing in different NHP species. The table for each home-cage approach highlights the relevant information characterizing each approach, covering: the components involved in executing the cognitive tasks, the type of reward system, the species tested with the apparatus, and the availability of automatization techniques for cage task training, management of experimental sessions, and identification of animals interacting with the apparatus, as well as the inclusion of a detailed list of the hardware and software parts employed to build the home-cage setup.

Method Article	Task Devices	Reward System	Primate Species	Automated Step Training	Session Automatization	Primate Recognition	Required Component List
[87] Butler & Kennerley, 2019.	Android tablet	Liquid reward	*Rhesus macaques*	No	No	Face recognition	Yes
[81] Calapai et al., 2017.	Touchscreen	Liquid reward	*Rhesus macaques*	Yes	Yes	Manual post-session identification	No
[89] Curry et al., 2017.	Touchscreen	Liquid reward	*Rhesus macaques*	Yes	No	Separation from conspecifics	Yes
[93] Evans et al., 2008.	Monitor + Joystick	Solid reward	*Tufted capuchin monkeys*	Yes	No	Separation from conspecifics	Yes
[92] Fagot & Paleressompoulle, 2009.	Touchscreen	Solid reward	*Guinea baboons*	No	Yes	RFID	No
[88] Fizet et al., 2017.	Touchscreen	Solid reward	*Rhesus macaques*	Yes	Yes	RFID	No
[95] Sacchetti et al., 2022.	Touchscreen	Liquid reward	*Rhesus macaques*	Yes	No	Separation from conspecifics	No
[84] Womelsdorf et al., 2021.	Touchscreen	Solid and liquid reward	*Rhesus macaques*	Yes	No	Separation from conspecifics	Yes
[98] Scott et al., 2024.	Touchscreen	Liquid reward	*Common marmosets*	Yes	Yes	Separation from conspecifics	Yes
[91] Martin et al., 2022	Touch-screen	Solid reward	*Multiple macaque’s species*	Yes	Yes	None	Yes

The feasibility of providing an automated, task-specific training phase that is adaptable to the individual learning capabilities of each NHP has facilitated the investigation and evaluation of a broad range of cognitive processes, through the provision of multiple cognitive tasks sequentially by exploiting different delivery approaches. Facilitating training and testing across multiple cognitive tasks may also offer ethical advantages, particularly in support of the 3R principle of reduction. Ideally, the automation and standardization of effective protocols for training and administering multiple tasks could allow each animal to participate in distinct research projects and experimental investigations concurrently, thereby potentially reducing the overall number of animals required across separate studies. Furthermore, the integration of computerized task batteries, designed for use in both NHPs and humans, with a home-cage implementation has shown shared task-related behavioral effects across NHPs and humans, tested with the same tasks [99,100,101,102,103,104]. These findings highlight the strong translational potential of the home-cage approach for investigating cognitive and motor domains and tasks commonly studied in humans [105,106,107,108,109,110,111,112,113]. Such tasks may serve as valuable tools for assessing both normal and pathological functioning, and for initially evaluating the efficacy of behavioral or pharmacological interventions in NHPs, with the potential for subsequent application in addressing cognitive and clinical deficits in humans. The Cambridge Neuropsychological Test Automated Battery (CANTAB) is a set of computerized cognitive tasks specifically developed to offer a broad-spectrum assessment of cognitive functions and designed for applicability across various animal models [109,111,114] and human subjects [115,116]. Given its potential translational value, several studies employing the home-cage approach have integrated the CANTAB tasks to evaluate short-term memory [111,114,117], executive and attentional functions [111,118,119,120], associative learning [114], and motivational responses to food reinforcement [111], ultimately confirming its applicability and comparative validity relative to human performance. Such studies typically employed time-limited daily task administrations, either presenting multiple tasks sequentially within the same day upon reaching predefined performance criteria or across separate daily sessions to prevent potential interference between tasks. The multi-task approach subsequently became widely adopted within home-cage setups, implemented either through time-limited [81,84,91,93,114,119,120] or full-day [83,88,89,92,121] administration schedules, with multiple tasks presented on the same or different days. In this framework, Calapai and colleagues [83] introduced an innovative strategy, which allowed NHPs at the beginning of each trial to freely select among cognitive, motor, and enrichment-oriented tasks from a battery of available options. This approach resulted in spontaneous and sustained animal motivation to engage with the apparatus, even within the freely structured framework of concurrent multiple-task performance.

Voluntary and prolonged execution of multiple tasks throughout the day represents a major advancement of the home-cage approach over classical neurophysiological methods. This strategy can substantially reduce the time required for experimental data collection by systematically training and testing primates on a continuous, 24 h schedule using large sets of tasks in parallel, without negatively impacting NHPs’ daily welfare or routine. Nevertheless, further refinements in automation are necessary to prevent such continuous NHP testing from generating comparably high time and resource demands on research staff, particularly when managing large groups of animals. To address simultaneous training and testing requirements for groups of NHPs housed in mixed indoor and outdoor enclosures, Fagot and Paleressompoulle [92] developed a home-cage setup installed within sheltered testing chambers located in the outdoor enclosure area, providing animals continuous (24 h/7 days) free access. Their setup included a built-in algorithm capable of fully automated data acquisition, controlling several crucial processes such as accurate automatic identification of the NHP during interaction with the apparatus, storing session progress information even if interactions were interrupted and later resumed by the NHP, and performing real-time computations of performance metrics necessary to transition the animals between tasks and/or from training to testing phases. Furthermore, the algorithm balanced task assignments among NHPs with different levels of engagement with the apparatus. Similarly, Fizet and colleagues [88] designed a home-cage apparatus for NHP groups, integrating an algorithm specifically developed for the remote management and monitoring of the entire experimental procedure involving multiple task batteries (Figure 2C). This algorithm continuously maintained a detailed database of each NHP’s task-specific learning stage, performance history across previous sessions, and achievement of progression criteria. This allowed real-time, trial-based selection of tasks or task stages to be individually presented for each NHP. Additionally, all collected information, task parameters, and experimental conditions were remotely accessible and adjustable by experimenters via cloud-based services. To achieve complete experimental automation, home-cage setups must autonomously identify NHPs interacting with the apparatus to ensure the correct tasks and experimental phases are presented. Currently, some home-cage setups lack automatic animal identification techniques, opting instead to temporarily separate primates into specific cages dedicated to the home-cage setup, isolating them from their co-housed conspecifics [84,86,89,90,93,98]. Alternative methods use video cameras to record animal interactions for subsequent manual identification [81,94]. However, setups more committed to automation have adopted RFID (radio-frequency identification) systems, which detect microchips implanted in the animals [82,88,92,121], enabling the apparatus to activate and precisely log interactions whenever the NHPs are nearby. More recently, alternative strategies employing facial recognition techniques have emerged [87,97], leveraging neural networks trained on NHP-specific facial imagery to accurately identify individual animals in real-time through camera recordings integrated into the experimental setup.

A potential limitation of the home-cage approach might involve the negative impact of increased freedom and reduced control over task execution compared to classical chair methods, potentially affecting engagement, performance stability within and between experimental sessions, and susceptibility to environmental and social distractors. However, behavioral analyses conducted on NHPs tested with the main home-cage setups previously described revealed consistently high engagement [81,87,92,95,121], measured as the number of trials performed, as well as stable performance across extensive periods involving different cognitive task batteries, both in time-limited and prolonged daily testing schedules [81,84,88,89,91,114,120]. Stepwise training procedures implemented to prepare animals for task execution further demonstrated efficacy, promoting task-specific learning and aiding in the early identification of animals unsuitable for the experimental design [81,93,94,95,98]. Sacchetti and colleagues [95] directly investigated the feasibility of the home-cage approach using a variation of the non-match-to-goal task (Figure 2B). According to task design, NHPs were required to use information regarding the stimulus selected in the preceding trial to make correct choices in subsequent trials, thus asking the animals to maintain sustained attentional engagement throughout the session. Additionally, in specific sessions, a cursor controlled autonomously by the computerized control unit performed selected trials, requiring the animals to passively observe and retain information about the computer-made choice for subsequent trial executions. Despite the considerable cognitive demands imposed by this task, NHPs demonstrated rapid learning, executed a substantial number of trials without interruptions, and maintained stable intra-session performance, even when continuously exposed to visual and auditory interactions with a co-housed conspecific. Consistent with these findings, the proximity and social presence of conspecifics occupying the same or adjacent spaces allocated to the apparatus did not negatively impact engagement or task performance [104,121,122]. Instead, conspecifics appeared to foster a supportive social context, facilitating indirect learning by observation [86]. This social enhancement, combined with the documented capability of home-cage setups to reduce stereotypic behaviors and lower cortisol levels [123], underscores the home-cage approach as an ideal experimental setting that simultaneously promotes scientific productivity and animal welfare. In free-living NHPs, foraging constitutes a core behavioral activity, and the opportunity to engage in voluntary, effort-based food acquisition within captive settings can effectively mimic the foraging behavior expressed in the wild [124]. In this context, cognitive testing via home-cage apparatuses in NHPs housed in outdoor enclosures has been shown to align patterns of resting and feeding time with those observed in wild populations, in contrast to conspecifics without access to cognitive testing [125]. This evidence, combined with findings indicating an intrinsic reinforcing effect linked to interaction with the home-cage apparatus, even when resources are otherwise freely available [126], underscores the value of these approaches as a robust form of environmental enrichment. Institutions such as the Primate Research Institute in Japan exploit cognitive testing as enrichment, equipping outdoor structures with home-cage devices placed atop high climbing frames, thus maximizing the resemblance to natural foraging scenarios [124]. Such dual utility of home-cage approaches for both enrichment and cognitive testing supports the 3R refinement principle, potentially leading to substantial alleviation of the stress induced by classical experimental procedures, as described in this and previous Sections, as well as the distress caused by housing environments that lack adequate stimulation and fail to allow captive primates to express their natural behavioral repertoire.

In the final Section of this review, we examine the progressive integration of cellular activity recording methodologies, which has culminated in recent seminal implementations for investigating key topics in cognitive neuroscience.

## 3. Advancing Neurophysiology with Wireless Recording: Insights into Naturalistic Behavior

In more recent years, there has been a rapid technological development that, thanks mainly to the increasing miniaturization capability of electronic components, has led to the development of wireless neural activity recording systems. The possibility of using home-cage training systems is then not only useful to accelerate and make the training more efficient for behavioral tasks but, combined with the possibility of using new wireless recording systems, makes it possible to ‘outsource’ from the laboratory setting the whole experimental process, from training to data collection. This opens up important new scenarios and interesting perspectives for the neurophysiology of behavior. On the one hand, this approach allows the use of classical experimental paradigms, such as tasks performed on a computer monitor, where specific independent variables can be carefully controlled and dissociated to target a particular cognitive process, while making at the same time the whole procedure more efficient, faster, stress-free for the animals, and risk-free for the researchers. On the other hand, it offers the prospect of being able to rethink experimental designs that go beyond the limits imposed by the laboratory setting and to study natural behaviour in its greater complexity, increasing the ecological validity of the findings. Of particular interest is also the possibility of introducing experimental designs that adopt both approaches, comparing results with the aim of reinterpreting old knowledge and generalizing it (for a review addressing these issues in detail, see [127]).

### 3.1. Wireless Recording Technologies in Behavioral Neurophysiology

Beyond the interesting possible future applications, it must be noted that at the moment, wireless recording technology is still in its early days, mainly due to major engineering challenges. Today’s most modern systems allow intracranial recordings to be made from an ever-increasing number of electrodes [128]; however, as the number of electrodes increases, so does the amount of data that are generated, which must be amplified, digitalized, and stored or transmitted directly. Some systems can store data directly on storage devices such as SD cards [129,130,131,132], which, however, offer limited storage capacity and must be retrieved at the end of the recordings by taking the animal from its home-cage. Systems using a wireless data transmission system [128,133,134,135], on the other hand, require a high transmission rate, high power consumption, and an efficient heat dissipation system, in addition to having a more limited spatial range depending on the number and power of the antennas used to gather the signal. The batteries themselves can be mounted directly on the device and be disposable or rechargeable [133,136]. Alternatively, recording devices can be powered using systems that carry energy wirelessly, dispensing with the use of physical batteries and the risks related to their use, but introducing the problem of possible interference with the transmission of the neural signal [134,137,138]. Recent technological developments have made it possible to move from bulkier devices that require the use of vests containing the various components to be worn by the animal [139,140,141,142] to miniaturized devices whose weight can be supported by the head of even smaller animals, such as marmosets. All these are important factors, and advantages and disadvantages must be taken into account when deciding to move from a restrained setting to a freely moving setting for neural recordings (for a detailed review, see [143]).

### 3.2. From Restraint to Free-Moving Paradigms: Neural Correlates Across Behavioral Domains in NHPs

Notwithstanding, the increasing number of studies using home-cage training and wireless recording technologies with NHPs demonstrates a growing interest in recent years in a paradigm shift toward the study of what is referred to as “natural” or “naturalistic” behavior [144,145]. Historically, the interest stems from the realization that certain behaviors could not be reproduced, and therefore studied, in any way within a laboratory setting.

A case in point is the study of vocalizations. NHPs are social animals, and many species living in large groups communicate through spontaneous vocalizations that classical operant conditioning mechanisms cannot reproduce. Some of the first studies to use wireless recordings were concerned with studying vocalizations in squirrel monkeys [146,147,148], comparing neural activity recorded in animals restrained on a monkey chair, where vocalizations were elicited by kainic acid injections, and in animals that were free to move around in a room, where vocalizations were instead spontaneous. The possibility of using a more ethological approach has made it possible, for example, to locate brain structures that are involved in vocalization-dependent neural processes [148]. More recent studies investigated the neural correlates of vocalizations in marmosets [149,150,151,152,153], a highly vocal species, discovering for example that neurons in the premotor cortex are involved in the control of natural vocal behavior but not in orofacial motor functions [154], or that activity in the PFC during vocal behavior is modulated both before a vocalization is heard and during the response, suggesting a social monitoring role necessary for vocal coordination during a conversation [155].

**Figure 3:**
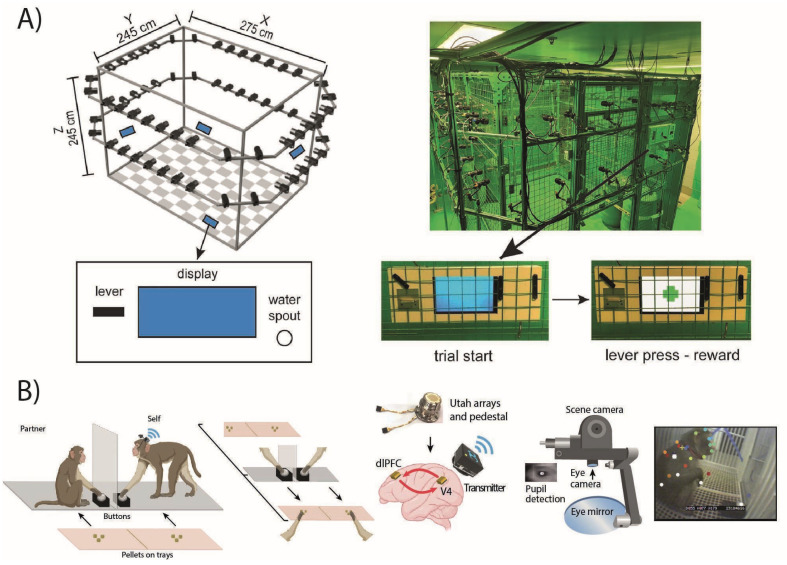
(**A**) Arena for the foraging task used by [164]. Cameras were used to track the position of the monkeys in a 3-dimensional space (x, y, and z axis) during wireless neural activity recordings. The feeding stations are depicted in blue and consist of a lever, a display showing a visual cue, and a valve for dispensing the reward. The monkeys were free to move around the arena between the different reward stations. Adapted from [164]. (**B**) Illustration of the cooperation task, neural recording device, and eye tracking device used by [201]. The two monkeys had to cooperate by simultaneously pressing a button to make the tray with the pellets move toward them. Wireless recordings were obtained from the prefrontal cortex and visual areas simultaneously. Pupil position and diameter were recorded via a portable wireless eye tracker mounted on the monkey’s head. Adapted from [201].

Another interesting area of application for studies using free-moving paradigms is the study of the neural correlates of spatial navigation. Freely moving paradigms that investigate spatial navigation have been widely adopted for electrophysiological recordings in species such as rodents, using tethered recording systems that rely on the use of suspended flexible cables that allow the animal freedom of movement in an open space. These systems, which have also been tested on small NHPs [149,156], however, may not be optimal for several reasons. First of all, in contrast to a constrained classical setting, they are not protected from possible manipulation by the animals that may damage them. Secondly, whereas animals such as rodents live and move in a predominantly two-dimensional space, NHPs, as arboreal animals, live in a three-dimensional space in which vertical movements are an essential part of their behavioral repertoire. Interestingly, the same issues apply to flying species, such as bats, where research to investigate spatial navigation using wireless recording systems has also begun to emerge [157]. Recent studies have used wireless recording to study neural activity during free movement in both marmosets and macaques. A strong focus has been directed toward the hippocampus, a highly studied structure in rodents and other species, and considered relevant for spatial navigation due to the presence of place cells, neurons tuned for specific regions in space [158,159]. Studies have shown common characteristics between rodents and NHPs, such as the presence of hippocampal place cells, although in different proportions [160,161], but also differences such as the limited role of theta oscillations, weakly correlated with place cell activity [162], or a greater number of neurons tuned for different spatial variables beyond the spatial place, such as head orientation and eye movement [130]. A recent study [163] that recorded the activity of hippocampal neurons from both marmosets and rats found that visual information has a greater influence on spatial navigation and place cell activity in marmosets, suggesting substantial differences between the two species, with NHPs using a navigation system that relies more on the visual system than rats. Navigation has also been investigated outside the hippocampus in other areas of the primate brain as for instance the PFC, an area that shows mixed selectivity for several navigational and non-navigational variables, such as reward information, in macaques engaged in foraging tasks (Figure 3A) in an open space [164,165], suggesting that the neural correlates of navigation are not restricted to the hippocampus and are part of more general cognitive processes that guide exploration, decision making, and action selection during natural behaviors. A recent study [166] explored in detail the neural representations of different types of actions in the motor and premotor cortex of the macaque, using a wireless recording system to compare the same neurons recorded both in a classical restrained laboratory setting, where the monkeys sat on a primate chair, and in a free-moving setting, where the monkeys were free to move in an open space. The authors found that neurons in the freely moving condition exhibited a richer and more diverse pattern of activity, simultaneously encoding multiple types of actions. Notably, even when comparing the same actions performed in both contexts—such as drinking or grasping food—only a small fraction of neurons maintained the same tuning across conditions (context-invariant neurons). Moreover, in the freely moving condition, these context-invariant neurons were also modulated by other action types to a greater extent than in the restrained condition, suggesting a more dynamic and integrative encoding of behavior and that action coding is strongly influenced by the context in which actions are performed. Data of this kind prove to be of extreme value and interest as they allow a direct comparison of the extent to which knowledge gathered over the years with the classical paradigms of the electrophysiology of behaviour can be generalized to natural behavior in the real world.

Another area of study made feasible by wireless recordings is the study of the neurophysiological correlates of sleep. When monkeys sit on a primate chair, the possibilities of studying sleep are limited, taking advantage of brief moments when the animal falls asleep spontaneously or using drugs for sedation [167,168,169]. Sleep, however, is composed of several phases of varying duration [170], and NHPs such as marmosets or macaques have a similar sleep pattern to humans, being predominantly diurnal animals that spend most of the night sleeping [171]. Recordings made in the home-cage make it possible to study the sleep–wake transitions that occur naturally between day and night, with recordings that can last up to many hours to cover the entire night [131,133,172,173,174].

One of the fields of application of wireless home-cage records with the most interesting prospects is certainly the study of social behavior [175]. Social neurophysiology with NHPs is a field that has received much attention over the past decades, with a multitude of studies that have helped shed light on the neural correlates underlying social interaction, focusing on the monitoring of others’ actions [176,177,178,179,180] and rewards [181,182,183], cooperation or competition [184,185,186,187], or the processing of others’ face, gaze and voice [188,189,190,191]. NHP neurophysiological studies provide information about the neural underpinnings of the many facets of social behaviors that would be difficult to study using other animal models. Nevertheless, the major limitations to studying social behaviour in a laboratory setting risk providing a picture that does not fully capture the breadth of the behavioral repertoire expressed by these animals in their natural contexts. These limitations include, for example, the difficulty in training two or more monkeys simultaneously to perform a joint task in which they must perform actions depending on what their partner did [192,193]. Some studies have overcome the difficulty by using interaction paradigms with a non-conspecific partner such as a human agent or even a virtual agent [194,195,196,197,198,199,200], which brings the advantage of being able to control the agent’s behavior directly, and thus the experimental variables under study, but with the disadvantage of moving away from an ecological context. Another limitation is that the restriction on a primate chair limits the ability to interpret a partner’s social cues, such as its body posture or gaze, and excludes physical contact. A recent study [201] investigated precisely the importance of visual cues during social cooperation by combining wireless recordings from the PFC and the visual cortex in two macaques in a free-moving context with eye movement recording (Figure 3B). The animals were able to see each other through a transparent divider and had to learn to cooperate by simultaneously pressing buttons to receive a food reward. The naturalistic setting plays a relevant role in being able to study how visual information influences social interactions, because it does not impose any kind of constraint on the animals during cooperation. The authors found that neurons from both areas encoded social visual information, with spike-coordination between areas that increased as monkeys learned to cooperate across sessions, and that the major contribution to the encoding of social variables came from couples of highly coordinated neurons. In this way, it was found that coordination between frontal and visual areas assumes a relevant role while learning to coordinate, suggesting, as with spatial navigation discussed before, that NHPs rely heavily on the sense of sight during many naturalistic behaviors, including social interaction. As stated earlier, physical interaction also plays an important role in macaque interactions in their natural environment, for example, through grooming, a social behavior that serves to establish bonds, determine hierarchies, and reduce stress.

Through wireless recordings directly from the home enclosure, Testard and colleagues [132] were able to study pairs of macaques during various natural social behaviors, both affiliative and aggressive. This ethological approach has allowed them to test far more behaviors than can be observed during a classic social interaction task, highlighting that many of these natural behaviors actually show much more distributed neural correlates across different brain areas than previously observed.

## 4. Conclusions

In this review, we gave an overview of the advantages of using a home-cage training system when studying the behavior of NHPs. In recent years, an increasing number of studies have been working on designing new home-cage training systems that can facilitate and improve both the work of researchers and the welfare of laboratory animals. The possibility of reducing stress, a central problem involving both ethical and scientific aspects in laboratories using NHPs, and, at the same time, facilitating and speeding up training are the main advantages that have led some laboratories to implement settings designed for home-cage training. All this opens up new scenarios that may represent the future of behavioral neuroscience, especially that of neurophysiology, thanks to technological development that has led to increasingly efficient wireless neural activity recording systems to be combined with home-cage training settings. NHPs have always been an essential model for biomedical sciences because of their evolutionary proximity to humans and their unique cognitive abilities in the animal kingdom. Macaque monkeys restricted merely to behavioral neurophysiology represent a pivotal animal model for studying a myriad of core processes of human behavior such as complex decision-making [202,203,204,205,206,207,208,209], working memory [210,211,212], attention [213,214,215,216], quantity and time perception [217,218,219,220,221], abstract representations [222,223], explorative–exploitative choices [224,225], motor control [226,227,228], dual-tasks performance [229], associative learning [230,231,232,233], and reinforcement learning [234,235,236]. At the same time, marmosets, whose small size and relative ease of housing can simplify procedures, are also emerging as viable models for many fields of study that have traditionally used macaques [237,238,239,240]. One of the most intriguing challenges for the future is the ability to critically reassess and refine traditional experimental paradigms that have long been used to study these cognitive processes. This involves not only questioning their underlying assumptions but also adapting and modernizing them to fit new contexts. In particular, there is a growing need to implement these paradigms within the framework of natural, everyday behavior, ensuring that they reflect how these cognitive processes, which have only ever been studied in an “artificial” laboratory setting, function in real-world scenarios. This approach necessitates accounting for species-specific behavioral patterns, exploiting the natural behavior of NHPs in natural contexts. By doing so, researchers can enhance both the ecological validity and the generalizability of their findings, ultimately leading to a deeper and more accurate understanding of cognition as it operates in natural environments [127].

The continued development of increasingly efficient wireless recording systems—capable of simultaneously capturing activity from a larger number of neurons across multiple brain regions—holds significant potential not only for basic research, such as investigating how neuronal ensembles coordinate to encode and transmit task-related information within and between neural circuits [241,242,243,244,245,246,247], but also for applied domains. These include the development of brain–computer interface (BCI) technologies for clinical use [248,249] and the advancement of biologically inspired AI and neurocomputational algorithms [250,251,252]. The hope is that in the future, the development of increasingly efficient wireless recording systems will enable the simultaneous acquisition of an increasing number of high-resolution neural data, which, combined with rapid innovations in deep learning, will foster and support new theoretical frameworks and analytical tools grounded in neurophysiological principles [252,253,254,255,256].

## Data Availability

No new data were created or analyzed in this study.
